# Supplementary material for: The Ionome–Hormone–Flavonoid Network Shapes Genotype-Dependent Yield Adaptation in Sugarcane
Source: Plants (Basel). 2025 Oct 16;14(20):3181. doi: 10.3390/plants14203181 (PMC12566931; doi:10.3390/plants14203181)
Supplement: Supplementary file 1 [file plants-14-03181-s001.zip › plants-3850953-supplementary.pdf]

Table S1 Precursor/product ion transitions and collision energies for Hormones in LC-MS/MS analysis.

Analytes	Scan mode	Precursor ions (*m/z*)	Product ions (*m/z*, **quantitative ion**/qualitative ion)	Collision energy (V)
1-Aminocyclopropanecarboxylic Acid	Positive	101.883	**56.217**, 73.883	13.54, 13.09
trans-Zeatin	Positive	220.05	**135.967**, 202.05	17.43, 12.38
Gibberellin	Negative	345.05	**221.05**, 239.05	23.75, 13.54
3-Indoleacetic Acid	Positive	175.967	**102.967**, 129.967	31.74, 14.86
Abscisic Acid	Negative	263.133	**152.967**, 219.05	9.75, 11.57
Salicylic Acid	Negative	136.883	**64.883**, 92.883	28.61, 15.82
Brassinolide	Positive	481.383	**349.217**, 445.383	13.64, 11.02

Table S2 Precursor/product ion transitions and collision energies for Flavonoid in LC-MS/MS analysis.

Analytes	Scan mode	Precursor ions (*m/z*)	Product ions (*m/z*, **quantitative ion**/qualitative ion)	Collision energy (V)
Salvianolic Acid B	Negative	717.133	**320.967**, 518.967	31.69, 16.42
Quinic acid	Negative	190.967	**84.883**, 92.883	20.92, 21.43
Caffeic Acid	Negative	178.883	**90.883**, 134.883	16.98, 11.98
Sinapic acid	Positive	225.05	**206.967**, 208.967	9.75, 15.46
Rosmarinic Acid	Negative	358.967	**160.883**, 196.883	17.89, 15.56
Rutin	Positive	611.217	**302.967**, 465.05	20.21, 11.42
Salvianolic Acid A	Negative	493.05	**184.883**, 294.967	25.83, 15.36
Astragalin	Negative	447.05	**254.883**, 283.883	38.31, 26.03
Coumalic Acid	Positive	141.05	**54.883**, 86.967	12.18, 9.25
Quercetin	Negative	300.967	**150.883**, 273.05	20.82, 16.12
